# Comparative clinical characterization of microflora between primary and secondary endodontic infections-An in vivo cross sectional study

**DOI:** 10.1016/j.jobcr.2025.09.018

**Published:** 2025-09-23

**Authors:** Mahantesh Yeli, Balaram Naik, RaghavendraD. Kulkarni, Vignesh Kamath, Prashant Moogi, Shruti Patil, Kishore Bhat

**Affiliations:** a Department of Conservative Dentistry and Endodontics, SDM College of Dental Sciences and Hospital, A Constituent Unit of Shri Dharmasthala Manjunatheshwara University, Dharwad, Karnataka, India; b Department of Microbiology, SDM College of Medical Sciences and Hospital, A Constituent Unit of Shri Dharmasthala Manjunatheshwara University, Dharwad, Karnataka, India; c Department of Prosthodontics Crown and Bridge, Manipal College of Dental Sciences Mangalore, Manipal Academy of Higher Education, Manipal, Karnataka, 576104, India; d Department of Orthodontics and Dentofacial Orthopedics, SDM College of Dental Sciences and Hospital, A Constituent Unit of Shri Dharmasthala Manjunatheshwara University, Dharwad, Karnataka, India; e Consultant Microbiologist, Arihant Superspeciality Hospital, Belagavi, Karnataka, India

**Keywords:** Primary lesions, Secondary infections, Anaerobic bacteria, Aerobic bacteria, PCR, Culture Tests

## Abstract

**Aim:**

The aim of the present study was to analyse the microbiota of primary and secondary/persistent endodontic infections of patients undergoing endodontic treatment with respect to clinical and radiographic findings.

**Materials and methodology:**

The experimental material included the samples collected from 139 patients. These subjects were divided into two groups of 74 and 65 patients. Group 1-Primary endodontic infections, Group 2-Secondary endodontic infections (Retreatment cases). The collected samples were subjected to aerobic and anaerobic bacterial cultures and selected specific uncultivable microorganisms were subjected to Polymerase Chain Reaction. The material collected in transport medium was mixed thoroughly and it was divided into two aliquots. One of the aliquots was used for aerobic culture and Polymerase Chain Reaction, the other was subjected to anaerobic culture study. Statistical analysis was done. P < 0.05 was considered as statistically significant.

**Results:**

For the given sample, the values for Porphyromonas gingivalis (p = 0.001), Prevotella intermedia (p = 0.017) and Streptococcus aerobic (p = 0.001), Klebsiella aerogens (p = 0.03) and Streptococcus (p = 0.005) and Dialister invisus (p = 0.024) showed statistically significant differences between primary and secondary lesions.

**Conclusions:**

The primary lesions showed significantly higher number of Klebsiella aerogens, Streptococcus, Dialister invisus and secondary lesions with Porphyromonas gingivalis, Prevotella intermedia, Streptococcus respectively.

## Graphical abstract

Graphical Representation of the methodology.

**Figure:**
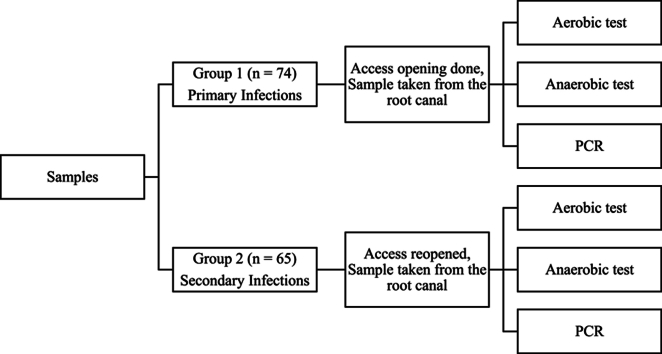

## 1 Introduction

The most common disease of the periradicular tissues is apical periodontitis which is an inflammatory disease and often characterized by invasion of oral pathogens invading the necrotic root canals, this was demonstrated by the classic study done by Kakehashi et al.^1^

There have been approximately 200–300 bacterial species which are present in the oral cavity but only few of them can be isolated from the necrotic root canals. Anaerobic bacteria were shown to dominate the microbiota of the untreated necrotic root canals like Peptostreptococcos, Prevotella, Porphyromonas, Fusobacterium, Eubacterium and Actinomyces and facultative aerobic Streptococci.^2^

The primary apical periodontitis or the primary endodontic infections usually heal once the root canals have been completely chemo-mechanically debrided and filled three dimensionally. Unsuccessfully treated root canals exhibit persistent inflammation called secondary apical periodontitis and often have distinct facultative anaerobes like Streptococcus, Lactobacillus and Enterococcus,^3^ and most of these bacteria have been known to resist conventional antimicrobial agents and disinfection procedures. Though the microbiome of primary and secondary endodontic infections has been studied in depth still there is uncertainty regarding their main etiological agents.^4^

Endodontic infections are polymicrobial in nature, with obligate anaerobes conspicuously dominating the microbiota in primary infection whereas Enterococcus faecalis and Candida albicans have been repeatedly identified from failed root canal treated cases.^5^ Endodontic infections have been studied by culture methods traditionally with reports suggesting over 50 % of oral microbiota still uncultivable, raising the possibility of many unknown endodontic pathogens. Species like Dialister invisus (81 %), Synergistes oral clone (33 %) and olsenella (33 %) have been detected in primary and persistent infections.^6^

Microbiota present in the primary and secondary endodontic infections associated with apical periodontitis were studied using 16SrRNA gene amplicon sequencing. The proportions of E. faecalis and F. nucleatum were respectively, higher and lower when comparing secondary to primary infected root canals. Though the microbiome of primary and secondary endodontic infections has been studied in depth still there is uncertainity regarding their main etiological agents. Endodontic infections have been studied by culture methods traditionally with reports suggesting over 50 % of oral microbiota still uncultivable, raising the possibility of many unknown endodontic pathogens.

The identification of bacterial taxa may provide the basis for targeted therapeutic approaches aimed at reducing the incidence of apical periodontitis.^7^ There is limited information available regarding characterization of endodontic microflora in root canals between primary infection and retreatment cases in our population.

A variety of newly identified uncultivable pathogens are found to be associated with endodontic infections. The most commonly found pathogens in endododontic infections are Dialister invisus, Synergistes oral clone BA121 and Olsenella uli. They are reported to be associated with persistent endodontic infections. This study tried to identify if these pathogens are present in the samples collected from our patients. Detection of uncultivated bacteria in infected root canals is the primary step in the studies leading to their role in the pathogenesis of endodontic infections or the pathogenesis of periradicular diseases. A large number of oral microbes are still not cultured successfully. To study these pathogens molecular techniques are essential. PCR is a commonly used molecular method to identify the uncultivable pathogens and has thus been used in the present study.

*Hence, the present study was conducted with the objective of characterizing microbiota present in primary and secondary endodontic infections (retreatment cases) associated with apical periodontitis and i*dentification and culture of commonly prevalent strains in primary and secondary infections of root canal and subjecting selected microorganisms for molecular methods. The Null Hypothesis taken into consideration for the study is that there is no significant difference in the characterization of endodontic micro flora in root canals between primary and secondary infection groups.

## 2 Materials and methodology

The present research is a cross sectional study design which was approved by the institutional ethical clearance committee (Ref.No-SDMIEC:86:2020).

The experimental material included the samples collected from the department of conservative dentistry and endodontics. The patients were screened for inclusion and exclusion criteria, explained about the study procedure and informed consent taken.

Group 1 (Primary endodontic infections) with inclusion criteria of symptomatic patients aged 18–60 years with single or multi-rooted non vital teeth with radiographic periapical radiolucency. However, patients with any systemic disease, pregnant and lactating women, tobacco users and patients who have been on any medications were excluded from the sample. Presence of more than 5 mm loss of periodontal attachment also was another excluding criteria.

Group 2 (Secondary endodontic infections/Retreatment cases) included symptomatic patients aged 18–60 years with single or multi-rooted non vital teeth with radiographic periapical radiolucency with a history of previous endodontic treatment of at least 2 years showing signs of failure. As in Group 1, once again patients with any systemic disease, pregnant and lactating women, tobacco users and patients who have been on any medications were excluded from the sample. Presence of more than 5 mm loss of periodontal attachment also was another excluding criteria.

Based on the calculation, the minimum sample size for statistical relevance was found to be 58. Those patients who met the inclusion criteria for Group 1, n = 74(Primary endodontic infections) and Group 2, n = 65(Secondary endodontic infections/Retreatment cases) were assigned to the respective groups.

Group 1: After plaque removal and rubber dam isolation, the tooth and the operative field were cleansed with 3 % hydrogen peroxide and then disinfected with 2.5 %NaOCL solution. The access was prepared with sterile endo access bur (MANI), the coronal pulp tissue from the infected tooth was removed mechanically and was placed into the transport medium. The working length was determined by radiographic method. The root canals were enlarged up to F1 size protaper rotary file till the working length, and the debris generated was transferred with help of # 25H File to the transport medium. Material from the canals was collected using sterile paper points which were then placed in the reduced transport medium (anaerobic culture) and equal amounts in TE buffer (aerobic culture and PCR). The Samples were sent to the central research lab for further molecular and microbiological analysis.

Group 2: The previous restoration was dismantled followed by removal of the Gutta Percha and the canal contents under rubber dam isolation. The root canals were enlarged up to F1 size protaper rotary file till the working length, and the debris generated was transferred with help of # 25H File to the transport medium. The gutta-percha and the debris was placed in the transport medium along with sterile paper points as explained before, the collected samples were subjected to aerobic and anaerobic bacterial cultures and PCR.

Anaerobic culture: The sample received in the transport medium Reduced Transport Fluid (RTF) was vortexed then inoculated on enriched and selective media according to the requirement within 24 h. For (Porphyromonas Gingivalis) Pg and (Prevotella Intermedia) Pi, the culture media included Blood agar as an enriched medium, Brucella agar with hemin and vitamin K and Kanamycin blood agar. The plates were incubated at 37 °C for 3–4 days in anaerobic jar as these are the obligate anaerobes. For (Fusobacterium Nucleatum) Fn, CVE (Crystal Violet Erythrocin) Agar was used as a selective medium and incubated anaerobically at 37 °C for 48–72 h. For (Aggregobacter Actinomycete comitans) Aa the Dentaid Agar was used as selective medium and the plates were incubated at 37 °C in 5–10 % CO_2_ jar for 48–72 h, as it is a facultative anaerobe requiring CO_2_ for optimum growth. At the end of incubation period the plates were examined to study colony characters. Colony count was also noted for proportion of various isolates and quantification. The identification of the isolates was done.

Aerobic culture: The transport medium received in the laboratory was vortexed to mix the contents thoroughly. The contents of the transport medium were inoculated on plates containing Blood agar, MacConkey agar, Brain heart infusion agar and Brain heart infusion broth as culture media. The plates were incubated in ambient air at 37^O^C for 48 h. At the end of the incubation period the plates were examined to study the colony morphology. The identification of the isolates was done using Gram stain, catalase, oxidase, motility and other biochemical reactions as per standard established protocols. All the aerobic isolates were subjected to sensitivity test.

PCR for selected organisms: The genomic DNA was isolated from each sample using phenol-chloroform method. The DNA was subjected to PCR assays using reported primers and protocols using conventional gel based PCR. PCR assays were set up for the following pathogens as they are frequently reported from endodontic samples. The primer pairs used for the pathogens were as follows:

1. Dialister invisus
  a. F - CAG AAA TGC GGA GTT CTT CTT CG
  b. R - CCC GGG AAC GTA TTC ACC G
2. Synergistes oral clone BA121
  a. AGA GTT TGA TCC TGG CTC AG
  b. TGC GAA AGG GTC GAT CCG C
3. Olsenella uli
  a. AGA GTT TGA TCC TGG CTC AG
  b. TGC GGC ACG GAG GGA TCG TCC C

## 3 Statistical analysis

The statistical analysis was done using IBM SPSS 20.0 Statistics (Version 20.0)(IBM Corp.,Armonk, NY,USA). Descriptive statistics like mean, SD, frequency and percentages were calculated. Number of colony forming units (CFU) between Primary and secondary was compared by Mann-Whitney test as the data did not follow the normal curve. Comparison of each microorganisms’ CFU between Primary and secondary lesion was done by Chi-square test. P value of less than 0.05 was considered as statistically significant for all comparisons.

## 4 Results

The study included 139 patients, with 74 presenting primary endodontic infections and 65 presenting secondary endodontic infections. Table 1 describes the descriptive and normative data of the various anaerobic organisms in the primary and the secondary lesions. There was statistically significant difference in the presence of microbiota in primary and secondary endodontic infection group for fewer organisms while others showed no differentiation between the two group of microorganisms. The results were verified through repeated sampling and cross-validation with independent laboratories. Table 1 illustrates the comparative microbial profiles of primary and secondary endodontic infections. On Comparison the microbial diversity in secondary infections was significantly greater than in primary infections (p < 0.05). The values for the Pg, Pi and Streptococcus were statistically significant in the secondary lesions when compared to the primary lesions. A strong positive correlation was found between the presence of specific anaerobes and the severity of symptoms in secondary infections. However there was no statistically significant difference with respect to Aa, Fn and Lb between the primary and secondary endodontic infection groups.

**Table 1:** Comparison of specific bacteria in primary and secondary lesions for anaerobic bacteria.

Type_of_lesion		N	Mean	Std. Deviation	Minimum	Maximum	Percentiles			MANN-WHITNEY TEST
							25th	50th (Median)	75th	P VALUE
**Aggregatibacter actinomycetemcomitans(Aa)**	Group 1	74	45.72	70.72	0.00	260.00	0.00	2.00	89.00	0.534
	Group 2	65	33.32	46.81	0.00	178.00	0.00	0.00	52.00	
**Porphyromonas gingivalis(**Pg)	Group 1	74	21.01	52.19	0.00	321.00	0.00	0.00	1.25	0.001^a^
	Group 2	65	42.71	78.33	0.00	360.00	0.00	4.00	36.00	
**Prevotella intermedia**	Group 1	74	49.82	86.58	0.00	400.00	0.00	0.00	55.25	0.017^a^
	Group 2	65	93.08	116.84	0.00	521.00	0.00	35.00	153.00	
**Fusobacterium nucleatum**	Group 1	74	26.72	73.12	0.00	400.00	0.00	0.00	2.00	0.415
	Group 2	65	24.74	61.63	0.00	310.00	0.00	0.00	16.50	
**Lactobacillus**	Group 1	74	13.51	35.63	0.00	195.00	0.00	0.00	0.50	0.30
	Group 2	139	22.73	55.64	0.00	354.00	0.00	0.00	4.00	
**streptococcus**	Group 1	74	33.15	85.92	0.00	450.00	0.00	0.00	2.25	0.001^a^
	Group 2	65	87.05	131.34	0.00	487.00	0.00	14.00	115.00	

a Statistically significant.

Table 2 describes the descriptive and normative data for the aerobic organisms in the primary and secondary lesions. A total of 23 aerobic organisms were cultured to assess their presence in primary and secondary endodontic lesions. Only with respect to two organisms, E aerogens and Streptococcus, there was statistical significant difference between primary and secondary endodontic lesions. The aforementioned aerobic microorganism were found to be significantly more in primary lesions compared to secondary lesions. However there was no statistically significant differences between the two group of lesions with respect to other aerobic organisms.

**Table 2:** Comparison of specific bacteria in primary and secondary endodontic infections for aerobic bacteria.

Microorganism	Type of lesion		Total	P-value
	Primary	Secondary		
Klebsiella aerogens	9.5 %	0.0 %	4.8 %	0.03^a^
Gram positive bacteria with spores	2.4 %	3.6 %	3.0 %	0.977
Klebsiella pneumoniae	3.6 %	2.4 %	3.0 %	0.977
Methycillin resistant Stahpylococcus aureus	1.2 %	0.0 %	0.6 %	0.800
Non fermentative Gram negative Bacteria	6.0 %	6.0 %	6.0 %	1.000
Gram positive bacilli	2.4 %	0.0 %	1.2 %	0.567
Coagulase negative Staphylococci	7.1 %	17.9 %	12.5 %	0.221
Acinetobacter spp	3.6 %	7.1 %	5.4 %	0.788
Citrobacter diversus	1.2 %	0.0 %	0.6 %	0.800
Streptococcus pyogenes	4.8 %	9.5 %	7.1 %	0.697
Pseudomonas aeruginosa	3.6 %	9.5 %	6.5 %	0.488
Enterococcus coli	2.4 %	4.8 %	3.6 %	0.875
Proteus vulgaris	1.2 %	0.0 %	0.6 %	0.800
Streptococcus	14.3 %	0.0 %	7.1 %	**0.00**5^a^
Moraxella catarrhalis	9.5 %	9.5 %	9.5 %	1.000
Micrococcus	1.2 %	1.2 %	1.2 %	1.000
Citrobacter freundii	2.4 %	1.2 %	1.8 %	0.952
Methicyllin resistant Coagulase negative Staphylococci	1.2 %	3.6 %	2.4 %	0.795
Candida	1.2 %	1.2 %	1.2 %	1.000
Enterococcus species	2.4 %	6.0 %	4.2 %	0.719
Enterobacter cloacae	1.2 %	2.4 %	1.8 %	0.952
Staphylococcus aureus	1.2 %	3.6 %	2.4 %	0.795
Enterococcus fecalis	0.0 %	1.2 %	0.6 %	0.800

a Statistically significant.

Table 3 describes the PCR done on the specific organisms like Dialister invisus, Olsenella uli and Synergistes. There was statistically significant difference with respect to Dialister invisus in the two group of lesions. Dialister invisus was found to be significantly higher in secondary compared to primary infections. However there was no statistically significant differences between Synergistes and Olsenella uli when compared with their respective primary and secondary infections.

**Table 3:** Comparison of specific bacteria in primary and secondary endodontic infections under PCR.

Microorganism	Type of lesion		Type of lesion		Total	P-value
			Negative	Positive		
Dialister invisus	Primary	Count	37	34	71	0.024^a^
		%	52.1	47.9	100	
	Secondary	Count	21	45	66	
		%	31.8	68.2	100	
	Total	Count	58	79	137	
		%	42.3	57.7	100	
Olsenella uli	Primary	Count	55	16	71	0.672
		%	77.5	22.5	100	
	Secondary	Count	54	12	66	
		%	81.8	18.2	100	
	Total	Count	109	28	137	
		%	79.6	20.4	100	
Synergistes	Primary	Count	62	9	71	0.402
		%	87.3	12.7	100	
	Secondary	Count	61	5	66	
		%	92.4	7.6	100	
	Total	Count	123	14	137	
		%	89.8	10.2	100	

a Statistically significant.

## 5 Discussion

The success rates for endodontic treatment range from 86 % to 98 %, making it a very predictable technique. The radiographic results of the treated tooth^8^ and clinical signs and symptoms are also used to determine if this treatment was successful or not. Numerous studies have examined the identification and detection of microorganisms in the root canal of teeth that were obturated.^9^,^10^^,^^11^ The main reason why endodontic treatment fails, even after careful endodontic procedures, is the persistence of microorganisms in the apical portion of the root canal.^12^ This may happen when endodontic instruments and irrigants cannot reach every area of the canal system and get rid of microbes efficiently^13^^,^^14^^.^

The root canal microflora between primary endodontic cases and retreatment cases differs. Literature Review showed distinct differences in the microbial profiles of primary and secondary endodontic infections. In the present study, the presence of specific aerobic and anaerobic microrganisms were assessed in primary and secondary endodontic lesions by various cultures. In the present study, we found that that there is statistically significant difference in the microbiota in primary and secondary infections. Secondary lesions had significantly higher microorganisms when compared to primary for Pg, Pi and STRE. However there was no significant differences between two groups for Aa, Fn and Lb.

The current study is in contradiction with the studies Kipalev et al.^15^, Sassone et al.^16^ which have concluded that the microbial composition of primary and secondary endodontic lesions differ significantly, with primary lesions generally showing a higher prevalence of bacteria such as Pg, Pi, and Fn. Study by Serge et al.^17^ has concluded that the Fn is found to be significantly lesser in primary endodontic lesions which is in contrast to our studies. Study by Pourhajibagher et al. ^18^ has concluded that the Aa is found to be more prevalent in primary endodontic infections compared to secondary. Our study found no significant difference with respect to Aa, Fn in contradiction to study by Gomes et al.^19^ who has found Aa, Fn to be more in secondary endodontic lesions. F. nucleatum was linked to wet root canals, haemorrhagic exudate, tooth decay, inadequate restoration, poor endodontic obturations, oedema, tooth mobility, spontaneous discomfort, sensitive to percussion, and pain upon palpation.

With respect to aerobic microorganisms, 23 aerobic microorganisms were cultured to assess their presence and E aerogens and Streptococcus spp were found to be significantly more in primary compared to secondary endodontic infections. This study is in agreement with study by Jose et al.,^20^ LM et al.^16^ Chavez et al.^21^ which concluded that Streptococci spp to be more prevalent in primary endodontic lesions. Obligate anaerobic bacteria make up the majority of the oral microbial flora, which is extremely diverse and complex. Due to technical constraints, the majority of these are either not yet grown or are challenging to cultivate.

The purpose of this study was to identify three new oral bacterial species—Dialister invisus, Olsenella uli, and Synergistes—using the quick and easy polymerase chain reaction (PCR) method and investigate their relationships to both acute and chronic endodontic infections. According to the study's findings, secondary infections had a noticeably greater D invisus than primary infections. However, there was no statistically significant differences when synergistes and Olsenella uli were compared with their respective primary and secondary infections. The conclusions of the present study are in agreement with the study by Shang et al.^22^ which has concluded that D. invisus, is the most prevalent species found in primary endodontic lesions of deciduous teeth. However, the study is in disagreement with the results by Christian et al. ^23^ which has concluded that D. invisus is found to be more in chronic apical abscess lesions.

A better knowledge of the microbiota involved in either primary or secondary endodontic lesions would help to more precisely define endodontic conditions predisposing to systemic diseases. Our study is the first of its kind to investigate the uncultivable three (D.invisus, olsenella uli and Synergistes) routinely found microorganisms in primary and secondary endodontic lesions in vivo for the Indian population. Variations in the microbiota of endodontic infections could be caused by the differences in the host and by environmental factors such as public health conditions, dietary habits, patient's immune response, and the weather in different geographic regions.

## 6 Conclusion

Within the limitations of the study, we could reach the conclusion that the microbiota of primary and secondary endodontic lesions vary significantly with greater microbial diversity in secondary infections. The secondary lesions had significantly higher anaerobic microorganisms when compared to primary infections with predilection for Porphyromonas gingivalis, Prevotella intermedia, Streptococcus and D. invisus. However, Primary lesions had significantly higher aerobic microorganisms when compared to secondary and showed increased predilection for E. aerogens and streptococcus spp. The conclusions derived from our study would have a great clinical significance as a thorough knowledge of identification of uncultivable organisms would help in the reducing the endodontic treatment failures and targeted therapeutic treatment approaches can be applied.

## Source of funding

This study was conducted without any external funding.

## Declaration of competing interest

The authors declare that they have no known competing financial interests or personal relationships that could have appeared to influence the work reported in this paper.
